# Good News for Nuclear Transgene Expression in *Chlamydomonas*

**DOI:** 10.3390/cells8121534

**Published:** 2019-11-28

**Authors:** Michael Schroda

**Affiliations:** Molecular Biotechnology & Systems Biology, TU Kaiserslautern, Paul-Ehrlich Straße 23, D-67663 Kaiserslautern, Germany; schroda@bio.uni-kl.de

**Keywords:** algal synthetic biology, Golden Gate cloning, modular cloning, algal biotechnology, transcriptional gene silencing, histone modifications

## Abstract

*Chlamydomonas reinhardtii* is a well-established model system for basic research questions ranging from photosynthesis and organelle biogenesis, to the biology of cilia and basal bodies, to channelrhodopsins and photoreceptors. More recently, *Chlamydomonas* has also been recognized as a suitable host for the production of high-value chemicals and high-value recombinant proteins. However, basic and applied research have suffered from the inefficient expression of nuclear transgenes. The combined efforts of the *Chlamydomonas* community over the past decades have provided insights into the mechanisms underlying this phenomenon and have resulted in mutant strains defective in some silencing mechanisms. Moreover, many insights have been gained into the parameters that affect nuclear transgene expression, like promoters, introns, codon usage, or terminators. Here I critically review these insights and try to integrate them into design suggestions for the construction of nuclear transgenes that are to be expressed at high levels.

## 1. Chlamydomonas reinhardtii—A Versatile Model System

*Chlamydomonas reinhardtii* is a unicellular green microalga living in the soil and in the pelagic zone of lakes [1]. *Chlamydomonas* has emerged as a valuable model organism for basic research e.g., on photosynthesis and chloroplast biogenesis [2], the biology of cilia and basal bodies [3], the cell cycle [4], the plant heat stress response [5], or the circadian clock [6]. Moreover, *Chlamydomonas* has received much attention because of its ability to produce molecular hydrogen [7] and lipids [8], both with promise as biofuels. Important developments for major research fields in life sciences have their origin in basic *Chlamydomonas* research. Prominent examples are the insights into the structure and function of cilia for the understanding of human diseases [9], the recently made connection between photoreceptors and the quenching of excess excitation energy in photosynthesis [10], or the discovery of channelrhodopsins, which have founded the field of optogenetics [11]. Many molecular tools have been developed for *Chlamydomonas* and these have been comprehensively reviewed previously [8,12,13].

More recently, *Chlamydomonas* has been recognized as a host for the production of high-value chemicals and high-value recombinant proteins [14,15]. For the latter, the *Chlamydomonas* chloroplast is a suitable expression platform with yields reported to range between 0.5% and 5% of total soluble protein [16]. The disadvantage of the chloroplast as expression platform is that recombinant proteins need to be purified from whole-cell extracts and will not be glycosylated. Transgenes expressed in the nucleus, however, can be targeted for secretion, allowing recombinant proteins to be glycosylated and secreted into the medium, from where purification can be achieved more easily [17,18]. First reports comparing the efficiency of secretion signals and introducing tags with serine-proline repeats to improve secretion by enhanced glycosylation have been published [19,20]. However, the major limitation of nuclear transgene expression in *Chlamydomonas* has been the low level of expression achieved. Here, I provide an overview to the history of nuclear transformation and the pitfalls encountered regarding the expression of nuclear transgenes. I review approaches taken by the *Chlamydomonas* community to understand the mechanisms underlying inefficient nuclear transgene expression and to overcome this problem. Finally, I try to integrate the insights gained into design suggestions for the construction of nuclear transgenes that are to be expressed at high levels.

## 2. Nuclear Transformation of *Chlamydomonas* Is Robust and Easy

Nuclear transformation of *Chlamydomonas* was established in the late 1980s. This was demonstrated by the successful complementation of mutant lines defective in the genes encoding argininosuccinate lyase and nitrate reductase with the respective wild-type genes [21,22]. In both reports, DNA was delivered with a particle gun, leading to the stable integration of multiple transgene copies into the genome with rather low transformation efficiency (max. 25 transformants per µg DNA) and low transformation frequency (1 × 10^−8^ to 2 × 10^−6^). The particle gun was soon replaced by a protocol based on the agitation with glass beads, which results in the stable integration of fewer transgene copies with higher transformation efficiency (500 transformants per µg DNA) and frequency (~2 × 10^−5^) [23]. Eventually, electroporation was robustly developed to deliver transgene DNA into *Chlamydomonas* nuclei, with transformation efficiencies of up to ~1.9 × 10^5^ transformants per µg DNA and a transformation frequency of 2.6 × 10^−3^ [24]. Protocols for both agitation with glass beads and electroporation require that cells lack cell walls, which is accomplished either by treating them with autolysin before transformation, or by using cell wall deficient mutants. The introduction of a square electric pulse-generating electroporator enabled electroporation also of walled *Chlamydomonas* cells with a transformation efficiency of up to ~4000 transformants per µg DNA and a transformation frequency of ~10^−3^ [25].

The first dominant selectable marker allowing direct transformation of any *Chlamydomonas* strain was the *Chlamydomonas CRY1* gene, which encodes a mutated version of the ribosomal S14 protein and confers resistance to the translation inhibitors emetine and cryptopleurine [26]. Soon, other dominant selectable markers followed, including the *ble* gene from *Streptoalloteichus hindustanus*, conferring resistance to phleomycin [27,28]; the eubacterial *aadA* gene, conferring resistance to spectinomycin and streptomycin [29,30]; the *aphVIII* gene from *Streptomyces rimosus*, conferring resistance to paromomycin [31]; or the *aph7″* gene from *Streptomyces hygroscopicus*, conferring resistance to hygromycin B [32]. Currently, the dominant selectable markers based on enzymatic activities (*aph7″*, *aphVIII*, and *aadA*) are the most widely used ones because they robustly allow for high transformation rates and do not have side effects like double strand breakages potentially induced by phleomycin.

With the exception of few hot and cold spots, the integration of transforming DNA was found to occur randomly [33]. Transforming DNA, like a resistance cassette, can be subject of endonucleolytic cleavage during transformation [30,33,34,35]. Moreover, its uptake by a cell may be accompanied by the uptake of DNA from lysed cells, which also can be cleaved by the endonuclease. The resulting mix of DNA fragments either is concatenated and inserted into one site, or the fragments are inserted at independent sites into the genome [29,33,35]. Frequently, deletions and inversions of genomic DNA flanking the insertion site of the transforming DNA occur [34,35,36,37]. It was proposed that large deletions occur more frequently when the transforming DNA is large, plasmid-derived, and delivered by agitation with glass beads as compared to when it is small, PCR-derived, and delivered by electroporation [36].

## 3. Mechanisms of Transcriptional Gene Silencing in *Chlamydomonas*

If a nuclear transgene is constantly under selection pressure, it will be expressed in *Chlamydomonas*. However, if the selection pressure is relieved, *Chlamydomonas* readily silences transgenes [29,38]. Moreover, transgenes may never be expressed, even if they are located next to the selection marker [39,40]. Unfortunately, in *Chlamydomonas* this is the rule rather than the exception and it is not known why transgene silencing is so efficient in this organism.

Transgene silencing in *Chlamydomonas* is caused by transcriptional or post-transcriptional mechanisms [29]. Post-transcriptional gene silencing usually is the result of inadequate transgene design, i.e., when no or too few introns have been inserted [41], the *Chlamydomonas* codon usage has not been respected [42], or when transgenes contain inverted repeats that give rise to double-stranded RNA [43,44,45,46]. Guidelines for proper transgene design are provided in Section 12 of this review. Transcriptional gene silencing is the main cause for transgene silencing in *Chlamydomonas* [38] and is largely mediated by protein factors that place specific histone modifications onto nucleosomes at the transgene loci to trigger the formation of a repressive chromatin structure—a mechanism that may have evolved to protect the genome from invading DNA [47,48,49,50].

**Figure 1:**
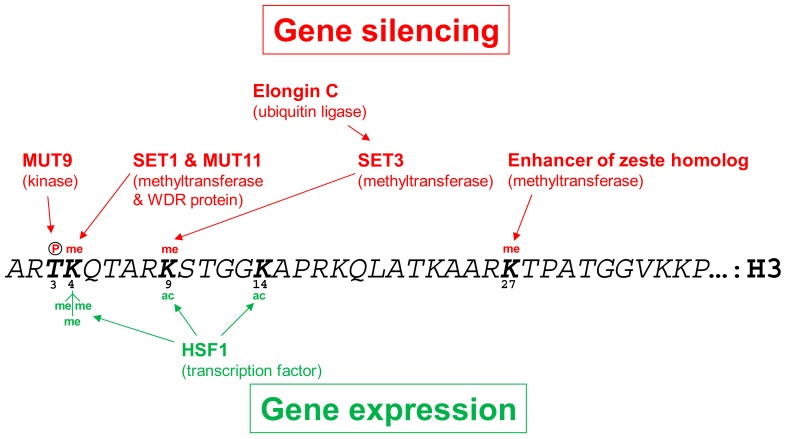
Effect of modifications at the N-terminus of histone H3 on gene expression in *Chlamydomonas*. Shown in black is the amino acid sequence of the unstructured N-terminus of histone H3 from *Chlamydomonas reinhardtii*. Letters in bold designate residues known to be modified with consequences on gene expression, with those shown in red on top of the sequence promoting gene silencing and those in green below the sequence promoting gene expression. me—methylation; ac—acetylation; P—phosphorylation. The protein factors involved in setting the modifications are indicated. See main text for details.

Nucleosomes consist of four different types of histones, H2A, H2B, H3, and H4, with each of them contributing two copies to form a histone octamer [51]. Especially histones H3 and H4 are subject of extensive post-translational modifications, largely occurring at their unstructured N-termini. These modifications constitute the histone code, which is read out by specific factors that recruit machineries for the remodeling of chromatin structure and thereby promote gene silencing or gene expression [52,53] (Figure 1). Histone marks that are known to occur on nucleosomes in promoter regions of silent genes in *Chlamydomonas* are histone H3 lysine 4 (H3K4) monomethylation [43,47,54], H3K9 monomethylation [42,43,45,49,55], H3K27 mono- and dimethylation [48], and H3T3 phosphorylation [47]. Histone marks generally found on nucleosomes at promoter regions of active genes are H3K4 trimethylation [48,49] and the acetylation of multiple lysine residues on histones H3 and H4 [42,43,45,48,49,56].

A likely scenario is that histones in nucleosomes forming de novo on foreign DNA upon its integration into the genome are specifically modified to tag and control this locus [49]. H3K9me1 has been found to be strongly enriched in nucleosomes on transgenic *Chlamydomonas* promoters, while this modification was virtually absent in nucleosomes on the respective native promoters. Hence, H3K9me1 is a good candidate for a modification set when new nucleosome arrays form on foreign DNA. Since the source of DNA (plasmid- or PCR-derived) had no influence on transgene silencing, it appears likely that the chromatin modifiers tagging foreign DNA are directly associated with the machinery involved in their integration into the genome and/or with the repair of double strand breaks [49]. The maintenance of the initial repressive marks during the cell cycle could then also be achieved by other chromatin modifiers. A lack of the initial modifiers is expected to have no consequences for cells kept in the laboratory but would render them more susceptible to invading DNA in the wild. The lack of modifiers that maintain histone modifications is expected to have more severe consequences, as the expression of regular genes would get deregulated.

Several factors involved in the maintenance and perhaps also the initial setting of repressive histone modifications have been identified in *Chlamydomonas* (Figure 1). One of them is MUT11, a homolog of the human WDR5 protein, which presents H3K4 for methylation [57]. In the *Chlamydomonas mut11* knock-out mutant, silenced single-copy transgenes and dispersed transposons get activated and the mutant is more sensitive to DNA damaging agents [58,59]. MUT11 was shown to interact with SET domain histone methyltransferases and RNAi-mediated suppression of SET1, a trithorax-like H3K4 histone methyltransferase, resulted in reduced levels of H3K4 monomethylation and the relief of silencing of a single-copy transgene and of *TOC1* retrotransposons [54].

Another silencing factor is the SU(VAR)3-9-related protein SET3. Suppression of SET3 by RNAi released the transcriptional silencing of tandemly repeated transgenes and correlated with a partial reduction of levels of monomethylated lysine 9 at histone H3 (H3K9), while repressed, single-copy euchromatic transgenes and dispersed transposable elements were not reactivated [55].

The MUT9 kinase phosphorylates threonine 3 at histone H3 and residues at histone H2A and is required for long-term, heritable gene silencing. In the *Chlamydomonas mut9* knock-out mutant, silenced single-copy transgenes and several transposons are derepressed and the mutant is more sensitive to DNA damaging agents. Reduced levels of H3T3 phosphorylation in *mut9* correlate with reduced levels of H3K4 monomethylation and suggest crosstalk between these histone modifications [47,59].

The *Chlamydomonas* enhancer of zeste homolog (EZH) catalyzes the methylation of lysine 27 at histone H3. RNAi-mediated suppression of *EZH* in *Chlamydomonas* resulted in a global increase in levels of histone H3K4 trimethylation and H4 acetylation, both characteristic for active chromatin, thus leading to the release of retrotransposons and of silenced, tandemly repeated transgenes [48].

Finally, the silencing of the transgenic *Rubisco small subunit 2* (*RBCS2*) promoter, driving the expression of an inverted repeat construct, was found to be associated with low levels of histone H3 acetylation and high levels of H3K9 monomethylation at the transgenic promoter [43]. Deletion of the *Elongin C* gene, which is a component of E3 ubiquitin ligase complexes, relieved silencing of the transgenic *RBCS2* promoter. The activated promoter was characterized by high levels of H3 acetylation and low levels of H3K9 monomethylation [45].

## 4. Expression Strains Allow Efficient Nuclear Transgene Expression in *Chlamydomonas*

All the above-mentioned factors involved in the maintenance/setting of repressive chromatin marks have been identified in an endeavor to understand the mechanisms underlying transcriptional gene silencing in *Chlamydomonas*. Mutants lacking these factors have not yet been exploited for the generation of expression strains. Exactly for this purpose, Neupert et al. performed a UV mutagenesis screen, using as starting point a transformant with a weakly expressed transgenic copy of the *Chlamydomonas CRY1* gene, conferring resistance only to low concentrations of emetine [50]. This strain (Elow47) was subjected to UV mutagenesis. The two resulting mutants, UVM4 and UVM11, were not only resistant to high concentrations of emetine, but also expressed another, newly transformed, heterologous transgene at high frequency to high levels [50]. Although it is not yet known which gene(s) is affected in the UVM4/11 strains, these data suggest that the affected factor(s) plays a role in the setting of negative chromatin marks at transgenic promoters. Importantly, the UVM4/11 expression strains have successfully been used for the high-level expression of various nuclear transgenes [20,41,42,60,61,62,63,64]. Therefore, they can be considered as an important breakthrough for basic research with *Chlamydomonas,* e.g., for the generation of overexpressing lines, and for promoting this organism as a workhorse for algal biotechnology.

Based on studies with *Volvox carteri* [65,66], Kong et al. suspected that a maintenance-type DNA methyltranferase (MET1) might be an additional silencing factor and indeed reported an increased frequency of high-level transgene expression in a *Chlamydomonas met1* insertion mutant [67]. With the goal to generate a super-expression strain, they started out from the *met1* mutant and subjected it to a similar UV mutagenesis screen as was used for generating the UVM4/11 strains [50]. Indeed, they obtained UV mutants that expressed transgenes with higher frequency and to higher levels than the UVM4 strain [68]. It will be interesting to know whether the same gene(s) is affected in UVM4/11 and the new expression strains.

## 5. Transcriptional Transgene Silencing Can Be Relieved to Some Extent by Specific Transcription Factors

All the factors described above mediate transcriptional transgene silencing in *Chlamydomonas* and, consequently, their inactivation resulted in the reactivation of transgenes (Figure 1). Interestingly, some factors can also actively counteract transgene silencing, at least to some extent. This is the case for the *Chlamydomonas HSP70A* promoter (abbreviated as *A* promoter). When transgene expression is driven directly by the *A* promoter, or when the *A* promoter is fused upstream of other *Chlamydomonas* promoters, like those from genes *RBCS2* (abbreviated as *R* promoter), *β_2_TUB*, or *HSP70B*, transgene expressing transformants were found at high frequency [39,69]. While transformation of the *ble* gene driven by the *AR* fusion promoter (*AR-ble*) construct resulted in about two-fold higher numbers of zeocin-resistant transformants than transformation with *R-ble*, *ble* mRNA levels in pools of zeocin-resistant transformants were about the same. This apparent contradiction was resolved in experiments where *AR-ble* and *R-ble* constructs were co-transformed with the *ARG7* gene and selection was on arginine prototrophy. Here, the fraction of co-transformants expressing *AR-ble* was more than three-fold higher than that expressing *R-ble*, indicating that the *A* promoter increased the fraction of expressing transgenes by counteracting transcriptional gene silencing [70].

In line with this conclusion was the observation that histones H3 and H4 in nucleosomes on transgenic *R* promoters contained higher acetylation levels, if the *R* promoter was preceded by an *A* promoter. Also, H3K4 trimethylation levels were higher, while H3K9 monomethylation was reduced [49]. While levels of H3K4 trimethylation at transgenic *R* promoters preceded by an *A* promoter were comparable to those at the native *R* promoter, levels of H3/4 acetylation were still below and levels of H3K9 monomethylation still far above those detected at the native *R* promoter. Hence, the *A* promoter alleviated transgene silencing, but did not fully overcome it [49].

Two regions within the *A* promoter were mapped that independently counteract *R-ble* transgene silencing, and the responsible *cis*-acting motifs were identified as heat shock element 1, TATA-box, and heat shock element 4 [49,70,71]. DNase I hypersensitive sites were detected on both motifs under ambient conditions, indicating the constitutive binding of a trans-acting factor [72]. This trans-acting factor turned out to be heat shock factor 1 (HSF1), as the inducible depletion of HSF1 relieved the activating effect of the *A* promoter on the *R* promoter in an *AR-ble* transgene [49] (Figure 1). HSF1 is the only canonical HSF of the two HSFs encoded by the *Chlamydomonas* genome and, since it forms trimers constitutively, HSF1 can potentially bind heat shock elements also under non-stress conditions [73]. Indeed, HSF1 was found to constitutively occupy the *A* promoter and was proposed to organize a scaffold, presumably containing mediator, TFIID, A, H, and E, that serve in recruiting RNA polymerase II to transcriptional start sites at the downstream promoter [49,56]. This scenario would explain why the spatial setting between *A* and *R* promoter is crucial for the activating effect of *A* [49,70,71]. Interestingly, upon the inducible depletion of HSF1, histone H4 acetylation at the *R* promoter in an *AR-ble* transgene declined with much slower kinetics than HSF1 occupancy and *ble* transcript abundance [49]. Hence, active chromatin marks like H4 acetylation per se are insufficient to promote promoter activity, it is the active recruitment of RNA polymerase that makes the difference.

## 6. Nucleosome Positioning and the Strength of Transcriptional Activators Might Affect Promoter Activity in Different Transgene Contexts

High-level transgene expression requires promoters that work robustly with all kinds of transgenes. In many organisms this is achieved with viral promoters that have evolved to overcome cellular constraints on the expression of foreign genes. Unfortunately, there are no reports on viruses infecting *Chlamydomonas*, perhaps because of the very efficient mechanisms for the silencing of foreign DNA sequences described above. Viral promoters commonly used in other organisms were at most only weakly active in *Chlamydomonas* [27,40,74,75,76,77].

Therefore, researchers went for native *Chlamydomonas* promoters driving genes whose gene products are known to accumulate at high levels. The outcome of this approach was rather heterogenous and an ill-understood dependence on the respective transgene construct was often observed. For example, a genomic copy of the α-tubulin gene was barely expressed in transgenic *Chlamydomonas* when it was driven by its own promoter, but strongly expressed when it was driven by the *R* promoter [78]. The *R* promoter was also functional in the context of the *ble* gene, even in the absence of selective pressure [27,28,70]. However, the *R* promoter was ineffective when it was supposed to drive expression of a genomic copy of the *HSP70B* gene, as was the native *HSP70B* promoter or the *β_2_TUB* promoter, while the *A* promoter worked well [39]. But the *β_2_TUB* promoter efficiently drove expression of the native arylsulfatase gene [79].

**Figure 2:**
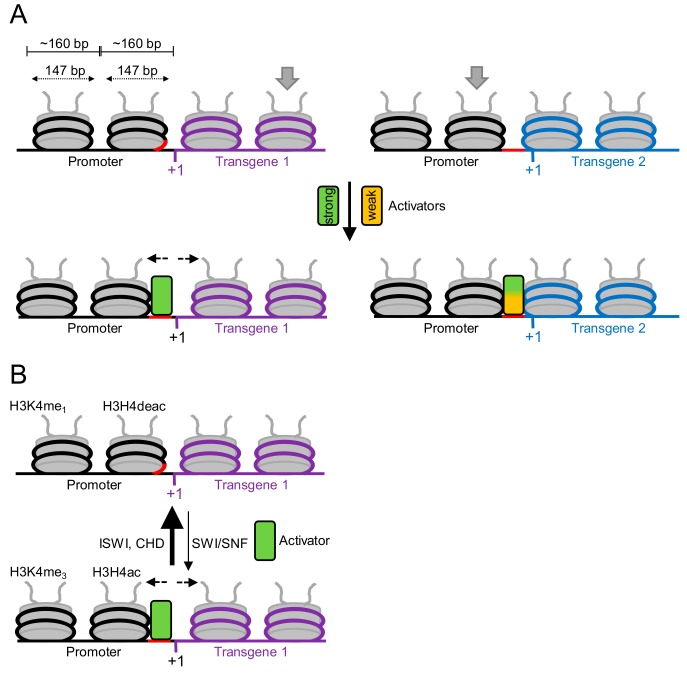
Hypothetical model explaining the different performances of transgenic promoters in *Chlamydomonas*. Shown is a nucleosome array formed on the promoter–transgene junction in the context of two different transgenes (purple and blue). (**A**) The positions of the nucleosomes in the array are influenced by transgene sequences such that *cis*-regulatory sequences (red) are inaccessible because covered by a nucleosome (transgene 1, purple), or accessible because located in the linking DNA (transgene 2, blue). Occluded *cis*-regulatory sequences can still be recognized by strong activators (green) that can alter nucleosome positions by themselves or by recruiting chromatin remodelers of the SWI/SNF-type. Weak activators (orange) can only bind to accessible *cis*-regulatory sequences in linker DNA. (**B**) Chromatin at transgene loci in *Chlamydomonas* is characterized by low levels of histone H3/4 acetylation and high levels of histone H3 monomethylation at lysine 4 (H3K4me_1_). Deacetylated nucleosomes are recognized by ISWI- and CHD-type of chromatin remodelers that consume ATP to evenly distribute nucleosomes and thereby occlude *cis*-regulatory sequences. In contrast, acetylated histones recruit SWI/SNF-type chromatin remodelers that consume ATP to slide away nucleosomes and expose *cis*-regulatory sequences. This allows access for activators (green). Modified from [80] and [81].

We learn that in *Chlamydomonas* the native promoter activity is not necessarily maintained in a transgene setting. This can be explained by the accessibility of *cis*-regulatory promoter sequences within the nucleosome array that has formed on the transgenic DNA (Figure 2). The covering of *cis*-regulatory sequences by nucleosomes may render them inaccessible to trans-activators and therefore results in an inactive promoter [80].

In the chromatin regions containing the *Chlamydomonas A* and *R* promoters, the nucleosome repeat length is ~160 bp [72]. With 147 bp of DNA wrapped around each nucleosome, only ~13 bp of linker DNA remain, i.e., the likelihood that *cis*-regulatory sequences at *Chlamydomonas* promoters are occluded by nucleosomes is high (Figure 2A). Different DNA sequences have different affinities for the histone octamer because the free energy required to bend the DNA sharply around the histone octamer depends on the sequence [82]. Moreover, N^6^-methyldeoxyadenosine (6mA) modifications were found to be enriched in linker DNA at transcriptionally active genes in *Chlamydomonas* and have been proposed to contribute to the precise positioning of nucleosomes [83]. Hence, if sequences and/or 6mA sites in the transgene determine nucleosome positioning, this could lead to unfavorable nucleosome positions on the transgene-driving promoter that render *cis*-regulatory sequences inaccessible (Figure 2A, top left). If sequences and/or 6mA sites in the promoter determine nucleosome positioning, *cis*-regulatory sequences are accessible and the promoter is active, independent of the transgene context (Figure 2A, top right).

A promoter may become independent of unfavorable nucleosome positions (and therefore of the transgene context) if it is served by a ‘strong’ *trans*-activator that can get access to *cis*-regulatory sequences even if they are covered by nucleosomes (Figure 2A bottom and Figure 2B). This is most likely achieved by the recruitment of chromatin remodeling factors of the SWI/SNF type that consume ATP to slide away or eject nucleosomes [52,80,84]. This activity is enhanced by acetylated histone H3. SWI/SNF remodelers have the ISWI/CHD-type of remodelers as counterplayers, which consume ATP to re-establish a dense nucleosome array, thereby promoting the occlusion of *cis*-regulatory sequences and thus gene silencing. The activity of these remodelers is enhanced by unmodified histone tails. Therefore, the low levels of histone acetylation frequently observed at transgenic promoters in *Chlamydomonas* [43,49] points to the occlusion of *cis*-regulatory promoter sequences by ISWI/CHD-like activities as one mechanism underlying transgene silencing in this organism.

Even for transgenes equipped with an efficient promoter, high variation in transgene expression levels is observed between different transformants generated with the same transgene [28,39]. This phenomenon is also referred to as position effect, as transgene expression is considered to be affected by the chromosomal integration site [85]. Position effects often make it necessary to screen dozens of transformants for a few with high expression levels. Moreover, they make it necessary to pool hundreds of transformants to make comparisons of promoter strengths meaningful [49,71]. Position effects were claimed to be overcome in the UVM4/11 strains [50], but this view was challenged previously [63]. A position effect was proposed to be due to the distance in 3D nuclear space that a transgenic promoter has to a nuclear region (‘factory’) containing the appropriate factors necessary for the transcription of that particular promoter [86]. This idea is attractive, but it does not explain why two transgenes harboring the same promoter and placed next to each other on the same piece of transgenic DNA are expressed at very different levels (our unpublished observation)—these promoters would have about the same distance to the next ‘factory’. Hence, more mechanisms must be at work that cause position effects. For example, nucleosomes formed de novo on the transgenic DNA might affect the accessibility of *cis*-regulatory sequences more at one transgene than at the other. Furthermore, if the initial setting of repressive chromatin marks on nucleosomes formed on the transgenic DNA upon its integration into the genome is a stochastic process, it might affect one transgene promoter more than another.

## 7. Tricks to Surmount Poor Nuclear Transgene Expression

As mentioned above, transgenes do get expressed in *Chlamydomonas* if selection pressure is maintained. This circumstance was exploited by using the foot and mouth disease virus (FMDV) 2A peptide [87,88,89]. The 2A peptide consists of 19 to 39 amino acids that mediate ribosome skipping during translation to give rise to two proteins originating from a single ORF. Most of the 2A peptide sequence remains fused to the C-terminus of the first protein product, whereas the following protein contains only one amino acid of the 2A peptide at its N-terminus (this is always a proline). Rasala et al. fused the ORF coding for the Bleomycin protein (Ble) in frame with the 2A peptide and the protein of interest. Since resistance to phleomcyin is based on the stoichiometric binding of Ble to the drug, high level Ble expression is required. This in turn also leads to high-level expression of the protein encoded by the second ORF. The first protein product on the common ORF must not necessarily encode a protein conferring resistance, it only needs to be well expressed. This, for example, is the case for the IFT25 protein when its gene contains all three native introns [76]. The 2A peptide-based system was used to co-express several proteins at high levels, including fluorescent proteins targeted to various cellular subcompartments, a secreted fungal xylanase, mCerulean-tagged α-tubulin, the Cpf1 endonuclease, squalene synthase, RBCS2, and FKB12 [67,76,87,88,89,90,91,92,93].

A small disadvantage of this system is that the efficiency of ribosome skipping varies for unknown reasons between transformants expressing different fusion proteins and even between different transformants expressing the same fusion protein. Therefore, unpredictable amounts of non-processed fusion protein accumulate in addition to the processed one [67,76,87,88,91,92]. Although the efficiency of ribosome skipping increased when the extended 2A peptide with 39 amino acids was used instead of the minimal 19-amino acids version, residual fusion protein was always detected [76,89]. The latter might give misleading results in localization studies, e.g., when the first ORF encodes Ble, which is targeted to the nucleus [94].

The use of bicistronic transcripts is an approach resembling the ribosome skipping approach but avoiding the accumulation of fusion proteins. Here, the ORF of the gene of interest (GOI) is placed on the same transcript upstream of the ORF encoding the APHVIII selection marker [95]. The stop codon of the former is separated by four nucleotides from the start codon of the latter (GOI-TAGccatATG-*APHVIII*). This setup leads to regular translation termination of the first ORF, followed by translation reinitiation at the close-by start codon of the second ORF [95]. The efficiency of this post-termination reinitiation is low, which is why it only works with the *APHVIII* marker that obviously confers resistance to paromomycin at low expression levels. With this system, paromomycin-resistant transformants accumulate the product of the GOI at high frequency to high levels, therefore reducing screening efforts.

## 8. Determinants of Nuclear Transgene Expression—Promoters

As outlined above, the activity of a *Chlamydomonas* promoter in a transgenic context may vary strongly with the transgene to be expressed. *Chlamydomonas* promoters that have robustly enabled constitutive high-level expression of several different transgenes and therefore must be served by strong *trans*-activators are the *AR* fusion promoter and the *PSAD* promoter [39,96]. While HSF1 is the strong *trans*-activator of the *AR* promoter [49], the identity of the *trans*-activator serving the *PSAD* promoter is not known. Although both promoters routinely showed strong activity, the *PSAD* promoter sometimes performed better than the *AR* promoter [62,97], sometimes no difference was observed between the two promoters [41,95], and sometimes the *AR* promoter outperformed the *PSAD* promoter [91,95,98]. These ambivalent results are likely due to ill-defined versions of the *AR* promoter used, since the stimulating effect by the *A* promoter depends on its length (it should comprise at least 467 bp upstream of the start codon) and its spatial setting versus the *R* promoter [49,70,71]. Moreover, some of these comparisons were done with an *AR* promoter harboring the first *RBCS2* intron close to the translational start site, which contains an enhancer sequence that would have stimulated also the *PSAD* promoter [41] (see Section 10). Finally, it is likely that the performance also of these promoters depends on the transgene context.

Several studies have compared the activities of other constitutive *Chlamydomonas* promoters with those of the *AR* and *PSAD* promoters as benchmarks. Again, these comparisons often used setups that introduced additional parameters, like different numbers/types of introns, different terminators, or ill-defined versions of the *AR* promoter, and often were limited to one or few reporters. For example, the *AR* promoter fusion employed by Lauersen et al. contains 20 additional nucleotides between both promoters when compared with the optimal fusion [70,71,99], and the *A* promoter employed lacks heat shock element 4, which increased expression of the *ble* transgene three-fold [49]. It is likely that both differences impair the fusion promoter’s performance. Hence, the outcomes of studies comparing promoter activities should be taken with caution. Nevertheless, the constitutive promoters analyzed displayed strong activities in the context of certain transgenes and are promising, especially to avoid repetitions of the *AR* and *PSAD* promoters in constructs harboring multiple transgenes. These promoters were the actin promoter [97], promoters *RPL23*, *RPL35a*, and *FDX1* [91], the *ARG7* promoter [100], and the *IFT25* promoter [76]. The latter could not drive expression of a GFP reporter gene to detectable levels but drove expression of its own gene (including all native introns) to very high levels and did so, too, when various ORFs were translationally fused downstream.

Several *Chlamydomonas* promoters have been reported to effectively drive the conditional expression of transgenes. The conditional expression of transgenes is desirable when the gene product creates a burden for cellular physiology. Conditional promoters are those from genes *CAH1* and *CAH4*, which are activated under low CO_2_ concentrations (air) in the light [101,102]; the *NIT1* promoter, which is inactive in the presence of ammonium in the medium and gets activated upon the exchange of ammonium by nitrate [103,104]; heat shock promoters like that of the *HSP70A* gene, which gets strongly induced upon a shift from 23 °C to 40 °C [39,89]; the *FEA1* promoter, which is induced by the depletion of iron from the medium by medium exchange, growth, or the addition of the iron chelator deferroxamine [92,93]; the *CYC6* promoter, which is induced by the addition of nickel or cobalt to the medium, or by the depletion of copper from the medium by medium exchange, growth, or the addition of the copper chelator TETA [105,106,107]; the *METE* promoter, which is repressed in the presence of vitamin B12 in the medium and activated in its absence [108]. Inducible expression of transgenes also can be achieved by including the riboswitch-containing *THI4* 5′ UTR between a constitutive promoter and the ORF of the transgene. Through riboswitch-mediated alternative splicing, the transgene’s ORF is translated only when thiamine (vitamin B1) is absent from the growth medium [109]. Note that the inducing cues are often associated with changes in cellular physiology that need to be accounted for. Moreover, since most *Chlamydomonas* ‘wild type’ strains cannot use nitrate as nitrogen source, the *NIT1* promoter cannot be used in these strains.

Like constitutive promoters, conditional promoters suffer from position effects. In our hands, position effects not only affect the maximum expression levels achieved for a transgene after induction, but also affect promoter tightness. For example, transgenes driven by the *NIT1* promoter tend to be expressed to some level even in the presence of ammonium [110]. Hence, extensive screening is necessary to identify transformants that do not express the transgene under repressive conditions but do so at high levels after induction. What might be causing the position-dependent leaky expression of conditional promoters? One possibility is that promoter activity is regulated at the level of chromatin structure, such that chromatin structure is remodeled by factors activated by the inducing signal which render *cis*-regulatory sequences accessible for general transcription factors. Such regulation might be impaired if the nucleosome array formed on the conditional promoters is dictated by DNA sequences surrounding the transgene integration site. Another possibility is that the transgenic conditional promoter gets located so close in the 3D nuclear space to a proper ‘factory‘ [86] that regulatory constraints are overridden.

## 9. Determinants of Nuclear Transgene Expression—Codon Usage

The first synthetic gene adopted to the codon usage of *Chlamydomonas* nuclear genes encoded GFP and was synthesized already in 1999. The codon-optimized *GFP* gene allowed for the first localization study employing a fluorescent protein in *Chlamydomonas* [94]. Codon optimization was stimulated by the finding that the *Streptoalloteichus hindustanus ble* gene with GC-content and codon usage similar to that of *Chlamydomonas* was stably expressed in *Chlamydomonas* [27,28], while the *aadA* gene with unbiased codon usage was poorly expressed and expression was unstable [29,38]. While the mechanisms behind this finding remained unresolved, it promoted the synthesis of more reporter genes with *Chlamydomonas* codon usage, including *Renilla* luciferase [111], *Gaussia* luciferase [74,112], NanoLuc [62], and several other fluorescent proteins [20,42,64,88,95]. Since gene synthesis has become cheap, the synthesis of all kinds of foreign genes with optimal *Chlamydomonas* codon usage has become routine [30,41,60,67,76,87,113,114,115].

In a systematic study to elucidate the mechanisms responsible for the improved expression of codon-optimized transgenes in *Chlamydomonas*, Barahimipour et al. employed four YFP-encoding sequences with different codon usage and GC-content, but driven by the same promoter and terminator [42]. It turned out that high-level YFP expression depended on an optimal codon usage, while a high GC-content itself was not enough. YFP protein levels correlated with transcript levels and, interestingly, also with active chromatin marks on nucleosomes at the promoter (higher levels of H4 acetylation and lower levels of H3K9 monomethylation). These findings indicate that transcripts with improper codon usage are less translated and therefore more prone to degradation. Degraded transcripts might then give rise to small RNAs that affect the chromatin state at the transgene locus via post-transcriptional gene silencing [116]. Definitely, codon optimization is crucial for high-level expression of nuclear transgenes in *Chlamydomonas* [117].

## 10. Determinants of Nuclear Transgene Expression—Introns

Introns can strongly increase gene expression in eukaryotes. This is achieved by two mechanisms. First, introns may contain enhancer sequences that increase rates of transcription initiation. Second, the mere presence of introns can increase transcript levels by a process termed intron mediated enhancement (IME). The mechanism proposed for IME is that efficient transcript elongation depends on the interaction of RNA polymerase II with the spliceosome. If this interaction is not provided, RNA polymerase aborts transcription and the immature transcript is degraded. By this mechanism, transcripts arising from real genes can be distinguished from those originating from the intergenic space or from invading DNA, both lacking introns [118]. With 7.3 introns per gene, *Chlamydomonas* genes are intron-rich relative to other unicellular eukaryotes and land plants; 92% of the genes contain introns with an average size of 373 bp, while the average size of exons is 190 bp [119]. Hence, primary transcripts in *Chlamydomonas* undergo intensive processing and there is evidence that enhancers and IME both contribute to gene expression in *Chlamydomonas*.

Clear evidence for the presence of an enhancer sequence in the first intron of the *RBCS2* gene was provided by the demonstration that this intron leads to higher transgene expression when placed upstream of the transgene driving *R* promoter in any orientation [28]. This enhancer appears not to be specific for the *R* promoter, because it enhanced expression of the *Pogostemon cablin* patchoulol synthase gene to a similar extent when it was driven by the *AR* promoter or the *PSAD* promoter [41]. Moreover, the first *RBCS2* intron also enhanced expression of the *Renilla* luciferase gene driven by the *CYC6* promoter when it was placed upstream of the promoter in 3′ to 5′ orientation [106]. Evidence for IME in *Chlamydomonas* comes from the finding that the second *RBCS2* intron also increased transgene expression although it lacks enhancer activity—it did not increase transgene expression when placed upstream of the *R* promoter [18,28,41,120].

Whether mediated by enhancers or IME, there is no doubt that introns dramatically increase transgene expression in *Chlamydomonas*, as the presence of introns has increased the expression of various transgenes when compared with transgene variants lacking these introns [28,32,41,62,70,76,95,106,120]. This was true also for transgenes driven by the *PSAD* promoter [41], although this promoter was proposed not to depend on introns [96]. In systematic studies, Baier et al. and Eichler-Stahlberg et al. inserted *RBCS2* introns into various codon-optimized transgenes and could show that transgene expression is enhanced by the regular insertion of introns (leaving exon sequences of <500 bp). Highest expression was achieved when two, or better, all three *RBCS2* introns were inserted in their native order [18,41]. These findings clearly demonstrate that intron engineering into transgenes is indispensable for high-level transgene expression in *Chlamydomonas*.

## 11. Determinants of Nuclear Transgene Expression—Terminators

Only few studies have investigated the role of gene terminator sequences for transgene expression in *Chlamydomonas*. However, these provide evidence that terminators have a strong impact: Kumar et al. detected up to ~40-fold differences in LUC reporter activity when the *LUC* gene was driven by the same promoter but different terminators. Here, the terminator of the *PSAD* gene performed best when compared to those of the *β_2_TUB* and *CCP1* genes [97]. Similarly, Lopez-Pas et al. found strong variation in *LUC* reporter gene expression depending on the terminator used [91]. Among the four terminators tested, those from genes *FDX1* and *RPL23* performed best. Given that the *RPL23* terminator contains an intron while the *FDX1* terminator does not, the strong performance of the *FDX1* terminator is remarkable. The lowest reporter activity was observed with the frequently used *RBCS2* terminator. This might be due to a promoter activity within the *RBCS2* 3′ UTR transcribing in antisense orientation and potentially giving rise to dsRNA that might initiate post-transcriptional gene silencing [31].

## 12. Design Suggestions for Nuclear Transgenes

For the construction of nuclear transgenes, I strongly recommend using the *Chlamydomonas* Modular Cloning (MoClo) kit [62]. This kit is available at the *Chlamydomonas* resource center and comprises 119 standardized genetic parts, i.e., promoters, coding sequences, tags, untranslated regions, etc. (https://www.chlamycollection.org/). These parts adhere to the syntax for Golden Gate-based modular cloning established for plant synthetic biology [121]. Using the Type IIS restriction enzyme BsaI and ligase, multiple genetic parts (level 0) can directionally be assembled into transcription units (level 1) in a single reaction. Using the Type IIS restriction enzyme BpiI and DNA ligase, several transcription units can then simultaneously and directionally be assembled into multigene constructs encoding entire metabolic pathways (level 2) [122].

**Figure 3:**
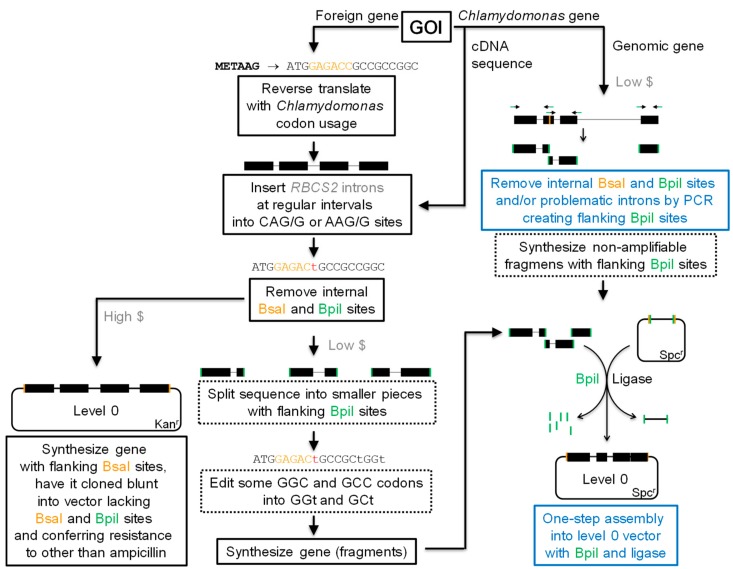
Workflow for the construction of nuclear transgenes as level 0 parts for Modular Cloning. Black text boxes indicate in silico steps or synthesis steps that are carried out by commercial companies. Blue text boxes indicate experimental steps that need to be conducted in the own laboratory and dotted text boxes indicate optional steps. Solid black boxes represent coding sequences of the gene of interest (GOI) and thin grey lines represent introns. Green and orange lines indicate recognition sites for Type IIS restriction enzymes BpiI and BsaI, respectively. Kan^r^—kanamycin resistance; Spc^r^—spectinomycin resistance. See main text for details.

Hence, for the construction of nuclear transgenes via the MoClo strategy, only a level 0 genetic part needs to be generated that contains the ORF of the GOI (Figure 3). If the GOI is a foreign gene, it needs to be synthesized de novo adopting the *Chlamydomonas* codon usage. For this, the amino acid sequence is reverse-translated using, e.g., the tool offered at http://www.bioinformatics.org/sms2/rev_trans.html [123] based on the *Chlamydomonas* codon usage provided by the Kazusa DNA Research Institute at https://www.kazusa.or.jp/codon/cgi-bin/showcodon.cgi?species=3055. Next, introns need to be inserted and internal BsaI and BpI recognition sites removed from the resulting sequence. We routinely insert the three *RBCS2* introns with regular spacing at CAG/G or AAG/G sequences (the slash is where the intron is). Although these flanking sequences differ from those in the *RBCS2* gene, they do correspond to the canonical sequences around *Chlamydomonas* introns [124] and match those determined experimentally to support efficient intron splicing [41]. Finally, it appears advisable to eliminate cryptic intron splice sites and to increase the folding energy of mRNA secondary structures around the start codon by introducing synonymous mutations into the designed sequence [117]. Commercial companies offer de novo synthesis and cloning of the designed sequence. In this case, the sequence should be flanked with BsaI recognition sites generating the four nucleotide overhangs assigned to that particular part, which depends on where in the final transcriptional unit it is positioned [121]. Moreover, it is required that the destination vector lacks internal BsaI and BpiI sites and confers resistance to an antibiotic other than ampicillin. These design steps can be performed conveniently with the recently released *Intronserter* software [120].

Companies offering gene synthesis at low cost (i.e., as linear, double-stranded, and non-clonal gene blocks) will complain about regions with very high GC content and repeats, which typically occur when using the optimal *Chlamydomonas* codon usage. This problem is somewhat alleviated with the three inserted *RBCS2* introns, which have a lower than average GC content (58% GC for the first and second intron, 61% GC for the third intron). The problem with repeats, e.g., resulting from the insertion of several copies of the same *RBCS2* intron, can be solved by splitting the GOI into several shorter pieces. In this case, each piece is synthesized independently with flanking BpiI recognition sites that generate unique four-nucleotide overhangs. The problem with very GC-rich regions can only be solved by editing several preferred GC-rich codons into less preferred ones containing A and T. In our hands, the editing of the third nucleotide of codons for Ala (GCC) and Gly (GGC) from C to T was tolerated, presumably because these codons are more common in *Chlamydomonas* genes than codons for other amino acids with T at the third position. Nevertheless, if maximum gene expression is the goal, this editing is not recommendable [42] and more expensive synthesis platforms should be consulted. The synthesized pieces of the GOI can be assembled into a level 0 vector in a single reaction step with BpiI and DNA ligase.

If the GOI is a *Chlamydomonas* gene with too many, too large, or too complex introns, it can be synthesized de novo based on the cDNA sequence with *RBCS2* introns inserted at regular intervals [41] (Figure 3). Internal BsaI and BpiI recognition sites again need to be removed. If the GOI is a *Chlamydomonas* gene with no or only few problematic introns (very long or with highly repetitive sequences), the gene can be amplified by PCR. If the sequence contains internal BsaI and BpiI recognition sites, these need to be removed for domestication [121]. This is achieved by introducing silent point mutations via primers covering the respective site that are flanked by BpiI recognition sites. This strategy can also be employed to remove problematic introns. Non-amplifiable pieces can be synthesized de novo as gene blocks, also with flanking BpiI sites (Figure 3). Using a MoClo destination vector for the particular part, several gene fragments can then be assembled directionally in a single reaction to yield the desired level 0 module [122].

**Figure 4:**
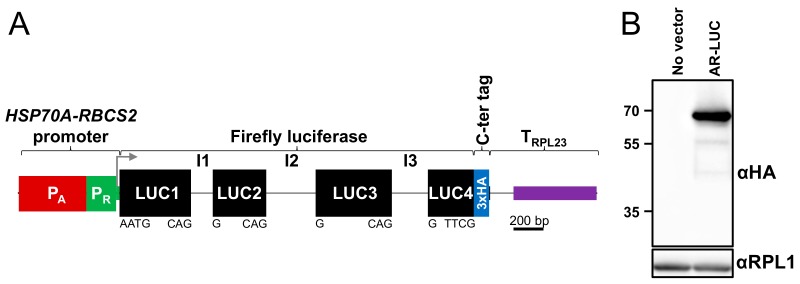
Example for a foreign gene encoding firefly luciferase that has been brought into the MoClo context for nuclear expression. (**A**) Level 1 construct. The red box depicts the *HSP70A* promoter harboring 467 nt upstream of the start codon in optimal spacing toward the *RBCS2* promoter (green box) harboring 217 nt upstream of its start codon. Black boxes represent the codon-optimized coding sequences of firefly luciferase (LUC) interrupted by *RBCS2* introns 1 to 3 (I1-I3, grey lines). Letters below are the respective flanking sequences. The blue box stands for a 3x hemagglutinin (HA) tag. The purple box indicates the 3′ UTR of the RPL23 gene, which contains an intron (grey line). The elements are drawn to scale. (**B**) Immunoblot analysis of the untransformed UVM4 recipient strain (no vector) and a UVM4 transformant harboring the *AR-LUC* level 1 construct. The expected mass of LUC-3xHA is 67.8 kDa. Figure courtesy of Miriam Schulz-Raffelt.

In our hands, nuclear transgenes designed with this workflow routinely give rise to high-level expression in *Chlamydomonas*. An example is shown in Figure 4. Here we have followed the left side of the workflow given in Figure 3 to generate a level 0 module encoding firefly luciferase harboring all three *RBCS2* introns. The level 1 construct consists of the optimal *AR* promoter, the luciferase ORF, a 3xHA tag, and the intron-containing *RPL23* terminator; the latter two are part of the current MoClo kit. This construct expressed the ~68 kDa foreign protein to the highest levels we have obtained until now.

## 13. Outlook

The combined efforts of the *Chlamydomonas* community have dramatically improved the capacity of *Chlamydomonas* to express nuclear transgenes at high levels. This has paved the path for this organism as a chassis for synthetic biology and biotechnology [14]. With the tools available, a properly designed transgene in our hands can achieve expression levels reaching those of highly expressed native *Chlamydomonas* genes. However, expression levels achieved from nuclear transgenes are still below those achieved by chloroplast expression or by other eukaryotic expression platforms like *Pichia pastoris* or insect cells using the baculovirus system. Hence, further improvements must be achieved by identifying and eliminating more factors mediating transgene silencing at the transcriptional and post-transcriptional levels to generate expression strains even better than UVM4/11. For example, we need to identify the methyltransferase(s) responsible for setting the H3K9me1 mark on nucleosomes forming on foreign DNA [49]. To this end, CRISPR/Cas mediated genome editing will be of great value [125,126].

Moreover, we need to further investigate the roles of regulatory sequences like promoters, enhancers, introns, and terminators and systematically analyze their performance in the context of various transgenes. To this end, the MoClo system is ideally suited, not only because it allows rapid cycles of construction and testing, but also because it promotes the exchange of defined, standardized genetic parts across the community [62].

Finally, a yet insufficiently investigated problem is the endonucleolytic activity on transforming DNA [33,35], which is likely to cause problems when constructs containing several genes are delivered. Such constructs can now easily be generated with the MoClo strategy [62]. Here, the events occurring upon the integration of such large constructs into the genome need to be investigated in detail.
